# Dr. Judson G. Kilbourne

**Published:** 1915-03

**Authors:** 


Dr. Judson G. Kilbourne, N. Y. University, 1884; died at his home in Utica, February 15. He was a member of the Oneida County Medical Society and was on the staff of St. Luke's Hospital, both of which organizations adopted appropriate resolutions, at special meetings.

